# Utility of the Swedish Anticholinergic Burden Scale in a memory clinic setting: a comparison with the Anticholinergic Cognitive Burden scale

**DOI:** 10.1038/s41598-025-34439-9

**Published:** 2026-01-06

**Authors:** Tanja Rube, Elisabet Londos, Per Johansson

**Affiliations:** 1 Cognitive Disorders Research Unit, Department of Clinical Sciences, Lund University, Malmö, Sweden; 2 Division of Clinical Geriatrics Department of Neurobiology, Care Sciences and Society, Karolinska Institute , Stockholm, Sweden; 3 Department of Clinical Sciences , Lund University , Helsingborg, Sweden; 4 Department of Internal Medicine , Sahlgrenska Academy University of Gothenburg , Gothenburg , Sweden

**Keywords:** Anticholinergic burden scale, Clinical pharmacy, Memory clinic, Muscarinic antagonists, Neurodegenerative disorders, Diseases, Health care, Medical research, Neurology, Neuroscience

## Abstract

Anticholinergic burden is associated with cognitive impairment, especially in older individuals with neurodegenerative disorders such as Alzheimer’s disease. The objective of the study was to explore the use of drugs displaying anticholinergic properties and examine the utility of the newly developed Swedish Anticholinergic Burden Scale (Swe-ABS) in a memory clinic setting. A descriptive cross-sectional study, involving individuals ≥ 50 years of age attending a memory clinic in southern Sweden between September 2017 and September 2021. In total, 657 individuals, mean age 72.4 ± 9.0 years, 375 (57.1%) males, were included. The study population used 4805 medications at an average of 7.3 ± 4.3 per individual. A total of 448 (68.2%) participants used drugs with any anticholinergic effects identified with the Swe-ABS of whom 179 (27.2%) had a high anticholinergic burden (i.e., Swe-ABS ≥ 3). The corresponding figures for the established Anticholinergic Cognitive Burden (ACB) scale were 314 (47.8%) and 97 (14.8%) respectively. The mean Swe-ABS score was significantly higher than the mean ACB score (1.7 and 1.0; *p* < 0.05). Individuals < 75 years had a significant higher Swe-ABS score and ACB score than individuals ≥ 75 years (Swe-ABS: < 75 years: 1.94 ± 2.20 and ≥ 75 years: 1.50 ± 1.54; *p* < 0.05; ACB: < 75 years: 1.13 ± 1.57 and ≥ 75 years: 0.80 ± 1.14 *p* < 0.05). Within the study population, a total of 64 drugs not listed in the Swe-ABS were identified. This study highlights the prevalence of drugs with anticholinergic effects among individuals presumably vulnerable to muscarinic antagonism. The Swe-ABS, a risk scale for ascertaining the anticholinergic burden, seems to effectively identify a substantial proportion of drugs commonly used on the Swedish market. Trial registration ClinicalTrials.gov, TRN: NCT03208569, Registration date: 27 June 2017.

**Supplementary Information:**

The online version contains supplementary material available at 10.1038/s41598-025-34439-9.

## Introduction

The inappropriateness of using drugs with significant anticholinergic effects (i.e., muscarinic cholinergic antagonists) in the treatment of older people has been known for decades^1^. This applies especially to individuals suffering from Alzheimer’s disease due to impaired cholinergic neurotransmission^2^. It has been suggested that the use of drugs with anticholinergic effects should be regarded as a potential modifiable risk factor for cognitive decline^3^.

The Swedish National Board of Health and Welfare (NBHW) has published quality indicators concerning potentially inappropriate medications (PIMs) for older people. They include long-acting benzodiazepines, tramadol, propiomazine, codeine, and drugs with anticholinergic effects, and are restricted to drugs with significant anticholinergic effects^4,5^. However, there are drugs displaying varying degrees of anticholinergic effects, contributing to the overall anticholinergic burden, referred to as the cumulative effect on an individual of taking one or more drugs with anticholinergic properties^6^. A high anticholinergic burden is associated with an increased risk of cognitive impairment and may be caused by many commonly used drugs not typically thought of as having anticholinergic effects^6–9^.

One method to assess anticholinergic burden in individuals is to use risk scales. These lists differ in the number and selection of drugs depending on prescribing patterns and drug availability, for example^10^. The Swedish Anticholinergic Burden Scale (Swe-ABS) was developed with the aim of being adapted to the Swedish healthcare system^11^. The Swe-ABS includes 381 drugs, of which 277 were rated as having no anticholinergic effects and 104 as having anticholinergic effects. For comparison the established Anticholinergic Cognitive Burden (ACB) scale includes 88 drugs with anticholinergic effects^7,11,12^.

Performing medication reviews for individuals aged ≥ 75 years with ≥ 5 medications to ensure the quality of drug treatment is mandated by NBHW. In addition, the guidelines include individuals with or at increased risk of drug-related problems regardless of age and number of medications^13^. Medication reviews, which include by clinical pharmacists, have been conducted for many years in Skåne County in different settings^14^.

A study from Australia showed that including clinical pharmacists in the multidisciplinary team can result in safer medication use in people with dementia living in residential aged care facilities^15^. However, even though pharmacists are increasingly being integrated into multidisciplinary teams, they are not typically involved in memory clinics^16,17^. In Sweden, pharmacists are involved in the pharmacological care of people with dementia in different healthcare settings^18,19^, although involvement in medication reviews at specialized memory clinics is scarce.

Furthermore, research into anticholinergic burden in patients attending memory clinics is limited^16^. One study conducted in a memory clinic that showed a reduction in anticholinergic burden scores after pharmacist-medication review intervention was found^17^. To the best of our knowledge, this is the first study to thoroughly investigate and describe the prevalence of drugs with any anticholinergic effects specifically in a memory clinic in Sweden.

The main objective of the current study was to examine the utility of the newly developed Swe-ABS in a memory clinic setting, and to describe the nature of the study population and their medication use at baseline, particularly drugs with anticholinergic effects. Additionally, the total anticholinergic burden according to the ACB and Swe-ABS scales was compared. The ACB scale was chosen for its established use, robust validation, and to enable comparability with previous studies^8–10^.

## Methods

### Study setting, design, and participants

This was a cross-sectional analysis of baseline data from a prospective observational study conducted at a memory clinic in southern Sweden, approved by the Regional Ethical Review Board in Lund (registration number 2016/856) and registered on Clinicaltrials.gov (TRN: NCT03208569). Individuals ≥ 50 years of age who had received a comprehensive medication reconciliation and medication review prior to the visit, attending their first visit between September 2017 and September 2021, were qualified for inclusion. Formal written informed consent to the study was mandatory. There were no stated exclusion criteria except for age < 50 years. Reasons for non-participation in the study were recorded during the first year; however, documentation was discontinued thereafter due to logistical constraints.

### Informed consent

Initial information about the study was given to the patient by the clinical pharmacist during a phone interview. Additionally, both oral and written information was given to the patient by a nurse at the first visit to the memory clinic. Only patients deemed being able to give an informed consent were invited to participate. Formal written informed consent was signed by the patient. In addition, consent was documented in the electronic medical record by the clinical pharmacist.

Symptom questionnaires completed by the patients and relatives were reviewed by the chief physician at the clinic to ensure that all patients were able to give informed consent.

### Medication reconciliations and medication reviews

Structured medication reconciliations were performed by clinical pharmacists and multiprofessional medication reviews were constructed in collaboration with physicians at the memory clinic.

An accurate list of each patient’s current medication was determined based on each patient’s prescription drugs (chronic and as needed), over the counter (OTC) medicines, herbal medicines, and dietary supplements. Information regarding medications was retrieved in the form of a questionnaire regarding current medication, electronic medical records, Medicine Check E-service^®^ (prescription repository) and a phone interview with the person responsible for the medication administration.

To categorize and prioritize drug-related problems, predetermined risk categories were used by the pharmacists. These included, for example, PIMs according to the NBHW, drug–drug interactions, as well as drug type or drug dosage related to renal or liver function. The current medication list, drug-related problems and subsequent recommendations were documented in the medical record by the clinical pharmacist. Identified potential drug-related problems and possible interventions were discussed by the physician and the clinical pharmacist. Any change in drug treatment took place in consultation between the physician and the patient.

### Anticholinergic burden classification

A medication was classified as anticholinergic if it had a score of ≥ 1 according to the ACB scale or the Swe-ABS. Additionally, PIMs with significant anticholinergic effects, as identified by the quality indicators published by NBHW, were presented.

In instances where the same substance was prescribed for both chronic and as-needed use, or appeared in multiple drugs, it was assessed only once. As-needed drugs, OTCs, herbal medicines, and dietary supplements were all considered in the assessment of the total anticholinergic burden.

### Descriptive statistics and subgroup analysis

Gender, age, medications, anticholinergic burden, PIMs, comorbidities at baseline were analysed. All patients obtained diagnosis according to International Classification of Diseases, 10th Revision (ICD-10), chapters F (Mental disorders), G (Diseases of the nervous system) or I (Diseases of the circulatory system). When these were not applicable diagnoses were given from chapters R (Symptoms, signs and abnormal clinical and laboratory findings, not elsewhere classified) or Z (Factors influencing health status and contact with health services). Descriptive analysis for quantitative data included mean, standard deviation, range, median, and Interquartile Range (IQR). The data were processed using Microsoft Excel software (Office 365, Microsoft, Redmond, WA, USA).

For the subgroup analysis, a parametric test, Student’s *t* test, was chosen despite the skewed distribution of anticholinergic burden in the study population. This decision was based on the relatively large sample size, which tends to mitigate concerns about normality, and to allow comparability with previous studies. The chi-squared test was used to find any differences in diagnoses at baseline between the age groups < 75 years and ≥ 75 years. The significance level of 5% (*p* < 0.05) was applied. All analyses were performed in IBM SPSS statistics (Version 29; IBM SPSS, Armonk, NY, USA).

## Results

In total, 663 individuals gave informed consent. Six individuals were excluded due to medical record blocked by one, while five did not complete the cognitive testing, leaving 657 study participants. During the first year, 78 potential individuals for the study were not enrolled. Of these, 28 individuals refrained from participating in the study, while 50 individuals were not approached when the inability to give informed consent was obvious.

Patients’ baseline characteristics and medication use are described in Table 1. The mean age was 72.4 ± 9.0 years and 375 (57.1%) were male.

Altogether, 4805 medications with an average of 7.3 ± 4.3 per individual were identified. The mean Swe-ABS score was significantly higher than the mean ACB score (1.7 and 1.0; *p* < 0.05). In total, 448 (68.2%) study participants used drugs with any anticholinergic effects identified with Swe-ABS of whom 179 (27.2%) had a high anticholinergic burden (i.e., Swe-ABS ≥ 3). The corresponding figures for the ACB scale were 314 (47.8%) and 97 (14.8%).

In 70 (39.1%) respectively 26 (26.8%) individuals the high anticholinergic burden was achieved solely by drugs with low anticholinergic potential (i.e., drugs with Swe-ABS and ACB = 1).

**Table 1:** Patients’ baseline characteristics and medication use.

Baseline	*n* (patients) = 657	*n* (drugs) = 4805
Age (years), mean (SD, range)	72.4 (9.0, 50–91)	
Male, *n* (%)	375 (57.1)	
Education		
≤ 9 years, *n* (%)	265 (40.3)	
10–12 years, *n* (%)	205 (31.2)	
≥ 13 years, *n* (%)	173 (26.3)	
Unknown, *n* (%)	14 (2.1)	
Number of medications per individual, mean (SD, range)	7.3 (4.3, 0–24)	
Swe-ABS, mean (SD, range)* Swe-ABS, median (IQR)	1.7 (1.9, 0–12) 1.0 (0–3)	
Patients with Swe-ABS 0, *n* (%)	209 (31.8)	
Patients with Swe-ABS 1, *n* (%)	179 (27.2)	
Patients with Swe-ABS 2, *n* (%)	90 (13.7)	
Patients with Swe-ABS ≥ 3, *n* (%)	179 (27.2)	
ACB scale, mean (SD, range)* ACB scale, median (IQR)	1.0 (1.4, 0–10) 0.0 (0–1)	
Patients with ACB 0**, *n* (%)	343 (52.2)	
Patients with ACB 1, *n* (%)	159 (24.2)	
Patients with ACB 2, *n* (%)	58 (8.8)	
Patients with ACB ≥ 3, *n* (%)	97 (14.8)	
Potentially inappropriate medications***tot, *n* (%) Drugs with anticholinergic effects, *n* (%)		122 (2.5) 70 (1.5)
Other, *n* (%)		52 (1.1)
Comorbidities, *n* (%)		
Cardiovascular	446 (67.9)	
Psychiatric	294 (44.7)	
Chronic kidney disease****	181 (28.0)	
Neurologic	173 (26.3)	
Diabetes	107 (16.3)	
Urologic	86 (13.1)	
Diagnosis F and/or G*****, *n* (%)	414 (63.0)	
Diagnosis I, R or Z*****, *n* (%)	243 (37.0)	

*p< 0.05 Student’s test, α = 0.05. ** ACB 0 indicates no drugs listed on the ACB scale. ***The Swedish National Board of Health and Welfare. ****Estimated glomerular filtration rate, GFR < 60 ml/min. Missing values n = 11. *****ICD-10. F (Mental disorder), G (Diseases of the nervous system), I (Diseases of the circulatory system), R (Symptoms, signs and abnormal clinical and laboratory findings, not elsewhere classified), Z (Factors influencing health status and contact with health services)

**Fig. 1:**
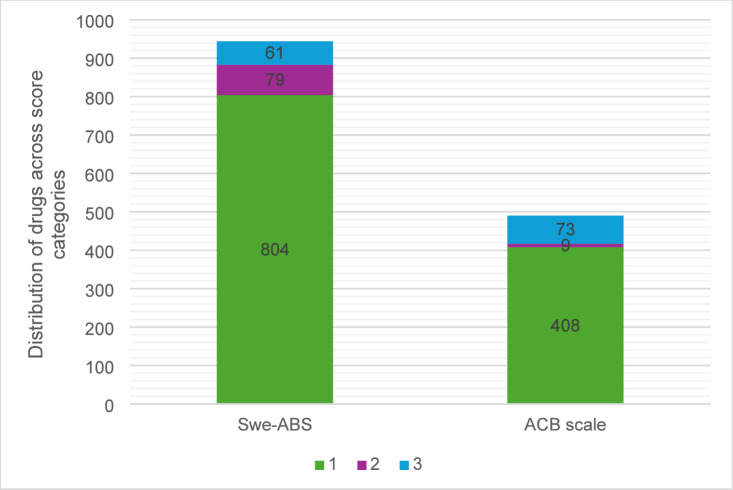
Distribution of drugs across score categories according to the Swedish Anticholinergic Burden Scale (Swe-ABS) and the Anticholinergic Cognitive Burden (ACB) scale.

Overall, 944 drugs with any anticholinergic effects were identified by the Swe-ABS, in contrast to 490 drugs according to the ACB scale (Fig. 1).

**Fig. 2:**
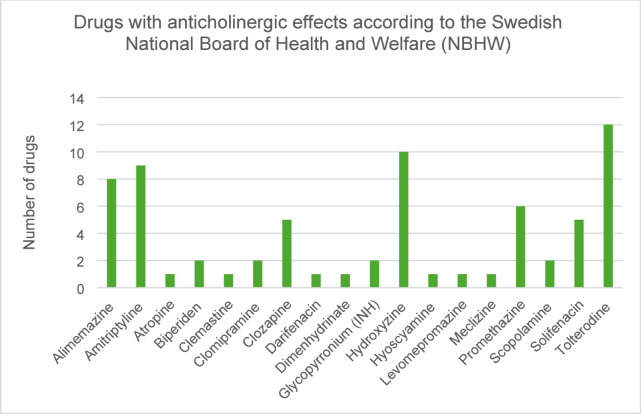
Identified drugs with anticholinergic effects listed in the guidelines concerning potentially inappropriate medications (PIMs) for older people published by the Swedish National Board of Health and Welfare (NBHW).

Together the study participants used 122 PIMs (Table 1) of which 70 were drugs with significant anticholinergic effects according to NBHW (Fig. 2).

**Fig. 3:**
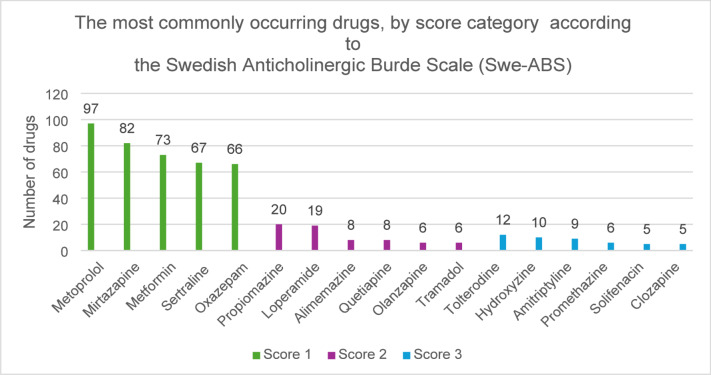
The most commonly occurring drugs in each score category according to the Swedish Anticholinergic Burden Scale (Swe-ABS).

**Fig. 4:**
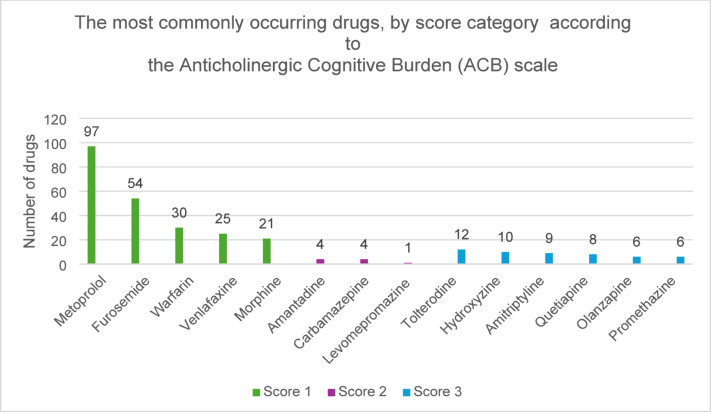
The most commonly occurring drugs in each score category according to the Anticholinergic Cognitive Burden (ACB) scale.

Tolterodine, hydroxyzine, amitriptyline, promethazine, solifenacin, and clozapine were the most commonly occurring drugs with definite anticholinergic effects according to the Swe-ABS (score 3). Propiomazine, loperamide, alimemazine, quetiapine, olanzapine, and tramadol were the most commonly occurring drugs with moderate anticholinergic effects (score 2) while metoprolol, mirtazapine, metformin, sertraline, and oxazepam were the most common drugs with low anticholinergic potential (score 1) (Fig. 3). Corresponding data according to the ACB scale: tolterodine, hydroxyzine, amitriptyline, quetiapine, olanzapine, and promethazine (score 3), amantadine, carbamazepine and levomepromazine (score 2), and metoprolol, furosemide, warfarin, venlafaxine, and morphine (score 1) (Fig. 4).

In the study population a total of 64 substances for parenteral or enteral administration could not be assessed, although authorized and available in Sweden, as they had not yet been included in the Swe-ABS (Additional Table 1).

### Subgroup analysis

Individuals < 75 years had a significant higher Swe-ABS score and ACB score than individuals ≥ 75 years (Swe-ABS: < 75 years: 1.94 ± 2.20 and ≥ 75 years: 1.50 ± 1.54; *p* < 0.05; ACB: < 75 years: 1.13 ± 1.57 and ≥ 75 years: 0.80 ± 1.14; *p* < 0.05).

There was no significant difference in the number of medications in the two age groups (Table 2).

**Table 2:** Mean anticholinergic burden according to the Swe-ABS and the ACB scale, by age.

	**Individuals (n)**	**Mean (SD)**	**Range**	**95% CI**	*p
Swe-ABS					
<75 years (mean: 66 y)	356	1.94 ± 2.20	0–12	1.71–2.17	
≥75 years (mean: 80 y)	301	1.50 ± 1.54	0–7	1.32–1.67	
					***<0.05***
ACB scale					
<75 years	356	1.13 ± 1.57	0–10	0.97–1.29	
≥75 years	301	0.80 ± 1.14	0–6	0.67–0.93	
					***<0.05***
Number of medications					
<75 years	356	7.16 ± 4.54	0–24	6.69–7.63	
≥75 years	301	7.50 ± 4.05	0–23	7.04–7.96	
					0.31

*Student´s t test, α = 0.05

Chronic kidney disease, cardiovascular and urological diseases were more common among individuals ≥ 75 years. Furthermore, this age group was more likely to get diagnoses involving central nervous system (F and/or G, ICD-10) at baseline (Table 3).

**Table 3:** Prevalence of diagnoses by age group (< 75 years and ≥ 75 years).

Diagnoses	< 75 years *n* = 356	≥ 75 years *n* = 301	*p
Cardiovascular, *n* (%)	224 (62.9)	222 (73.8)	***< 0.05***
Chronic kidney disease**, *n* (%)	46 (13.1)	135 (45.6)	***< 0.05***
Diabetes, *n* (%)	53 (14.9)	54 (17.9)	0.29
F and/or G***, *n* (%)	187 (52.5)	227 (75.4)	***< 0.05***
Neurologic, *n* (%)	83 (23.3)	90 (29.9)	0.05
Psychiatric, *n* (%)	163 (45.8)	131 (43.5)	0.56
Urologic, *n* (%)	33 (9.3)	53 (17.6)	***< 0.05***

*Chi-squared test, α = 0.05. **Estimated glomerular filtration rate, GFR < 60ml/min. Missing values n = 11 (< 75 years n = 350, ≥ 75 years n = 296). *** ICD-10. F (Mental disorder), G (Diseases of the nervous system)

## Discussion

In the present study, the utility of the newly developed anticholinergic risk scale, Swe-ABS, was examined in a memory clinic setting. The prevalence of anticholinergic drug use of 68.2% (the Swe-ABS) and 47.8% (the ACB scale) correlates with studies in similar settings. A study conducted in older people’s mental health services in the UK showed that 66.0% were prescribed drugs with anticholinergic effects determined by the Anticholinergic Effect on Cognition (AEC) scale^20^ and research from memory clinics in Australia showed a prevalence of 44.7% according to the ACB scale^16^. Moreover, a study conducted in nursing homes in Europe reported a prevalence of 53.8% according to the ACB scale and 29.4% according to the Anticholinergic Risk Scale (ARS) in people with dementia^21^.

The prevalence of anticholinergic drug use according to the Swe-ABS in the present study was higher than seen in studies conducted in primary care^22^ and in an unselected population sample^23^. In contrast, a study from the Rhode Island Hospital (RIH) reported a prevalence of anticholinergic drug use of 63.0% determined by the ACB scale, which is 15% higher compared with this study. The sample from RIH also had a higher mean ACB score of 1.5 compared with 1.0 in this sample^24^. The prevalence of anticholinergic drug use most likely varies with different settings and risk scales used to quantify anticholinergic burden^25^. The mean Swe-ABS score was significantly higher than the mean ACB score (1.7 and 1.0; *p* < 0.05), likely reflecting both the greater number of drugs included in the Swe-ABS and its adaptation to the Swedish market.

Previous findings show that drugs possessing weak anticholinergic effects are the main contributors to an overall high anticholinergic burden^26^. In this study, 27.2% of the study sample had a high anticholinergic burden ≥ 3 (stated by Boustani et al. as a clinically relevant cut-off)^7^ as determined by the Swe-ABS, while corresponding figures for the ACB scale were 14.8%. This finding is consistent with previous studies in similar samples^16,20^. In 70 (39.0%) respectively 26 (26.8%) individuals the total cumulative high burden was achieved only by drugs with low anticholinergic potential (i.e., drugs with Swe-ABS score and ACB score = 1). Consequently, the risk of unintended anticholinergic effects increases. Drugs with low anticholinergic potential are not included in the list of drugs with anticholinergic effects published by NBHW.

Among the important discrepancies between the ACB scale and the Swe-ABS is that several of the most common drugs identified by the Swe-ABS (tramadol, mirtazapine metformin, oxazepam, sertraline and propiomazine) are not listed in the ACB scale, while furosemide was assessed as having no anticholinergic effects in the Swe-ABS. However, it was the second most commonly occurring drug with low anticholinergic potential in the ACB scale. The inconsistencies may reflect the methodology used to develop the scales, which is partly based on expert opinions and may naturally lead to different results. They may also be influenced by variations in drug availability and prescribing patterns^10^, as well the fact that the Swe-ABS was developed in 2023 while the ACB scale was created in 2008 and updated in 2012.

Individuals < 75 years in this study had a level of anticholinergic burden which exceeded that of individuals ≥ 75 years according to the Swe-ABS and the ACB scale despite the fact, chronic kidney disease, cardiovascular and urological diseases were more common among individuals ≥ 75 years. This contradicts previous studies that showed a higher anticholinergic burden in older individuals^22,23,27^. This apparent inconsistency may be a result of the persistent work with multiprofessional medication reviews for individuals ≥ 75 years since 2011 in Skåne County. Reducing the number of drugs with significant anticholinergic effects, according to NBHW, has been a central part in the medication reviews, and annual evaluations show a decrease of defined daily doses between 2013 and 2025 in Skåne County^28^. This indicates the importance of considering side effects of anticholinergic drug use even in younger individuals.

The drugs identified in the study material, not currently included in the Swe-ABS, are in general drugs that appear to lack anticholinergic properties (Additional Table 1). Nevertheless, they require further evaluation and will be incorporated into future version of the Swe-ABS.

### Strengths and limitations

To assess the anticholinergic burden in a patient, an accurate medication list is essential. Clinical pharmacists have been involved in the multidisciplinary teams at the memory clinic since 2010 and are, hence, well integrated. Structured medication reconciliations and medication reviews were performed by clinical pharmacists presumably resulting in an increased accuracy. 

There are limitations worth mentioning. Neither the Swe-ABS nor the ACB scale encompasses every substance. Besides, they do not take dosage into account. Moreover, to ensure a complete assessment of potential drug exposure, even as-needed drugs seldom used were included in the total number of medications per individual, and no cut-off value based on usage frequency was applied. This may have overestimated the anticholinergic burden.

Another limitation is the high possibility of selection bias due to inability to give informed consent.

## Conclusions

This study highlights the prevalence of drugs with any anticholinergic effects among individuals presumably vulnerable to muscarinic antagonism. The Swe-ABS, a risk scale for ascertaining the anticholinergic burden, seems to effectively identify a substantial proportion of drugs commonly used on the Swedish market. These findings support the use of the Swe-ABS in clinical practice and encourage further research.

## Supplementary Information

Below is the link to the electronic supplementary material.

Supplementary Material 1

## Data Availability

The datasets used and analysed during the current study are available from the corresponding author on reasonable request.

## Abbreviations

ACB: Anticholinergic Cognitive Burden scale
ICD-10: International Classification of Diseases, 10th revision
NBHW: The Swedish National Board of Health and Welfare
OTC: Over the counter
PIMs: Potentially inappropriate medications
Swe-ABS: Swedish Anticholinergic Burden Scale
