# Supplementary material for: Utility of the Swedish Anticholinergic Burden Scale in a memory clinic setting: a comparison with the Anticholinergic Cognitive Burden scale
Source: Sci Rep. 2026 Jan 6;16:796. doi: 10.1038/s41598-025-34439-9 (PMC12780036; doi:10.1038/s41598-025-34439-9)
Supplement: Supplementary file 1 — Supplementary Material 1 [file 41598_2025_34439_MOESM1_ESM.docx]

# **Additional file**

**Table 1** Drugs authorized and available in Sweden and administered parenterally or enterally not included in the current version of the Swedish Anticholinergic Burden Scale (Swe-ABS)

| Acamprosate |
| --- |
| Alfacalcidol |
| Alfuzosin |
| Alginic acid |
| Alprostadil |
| Atomoxetine |
| Bendroflumethiazide |
| Betamethasone |
| Bictegravir |
| Botulinum toxin type A |
| Buserelin |
| Canagliflozin |
| Carbidopa |
| Cinacalcet |
| Cinchocaine |
| Dapagliflozin |
| Denosumab |
| Dexamfetamine |
| Dexpanthenol |
| Docusate |
| Dronedarone |
| Dutasteride |
| Efavirenz |
| Emtricitabine |
| Ephedrine |
| Eplerenone |
| Ethylmorphine |
| Etilefrine |
| Evolocumab |
| Lanthanum carbonate tetrahydrate |
| Letrozole |
| Linagliptin |
| Liraglutide |
| Mercaptopurine |
| Mirabegron |
| Netupitant |
| Niacin |
| Noscapine |
| Ondansetron |
| Palonosetron |
| Pancreas powder |
| Papverine |
| Pembrolizumab |
| Phenoxymethylpenicillin |
| Pomalidomide |
| Prasterone |
| Prasugrel |
| Pregabalin |
| Ranibizumab |
| Rasagiline |
| Riboflavin |
| Riluzole |
| Sodium picosulfate |
| Sodium polystyrene sulfonate |
| Tacrolimus |
| Tenofovir |
| Ticagrelor |
| Tinzaparin |
| Trabectedin |
| Tranexamic acid |
| Triptorelin |
| Valaciclovir |
| Valeriana officinalis (valerian) dried root |
| Zoledronic acid |
